# The Inflammatory Response to Double Stranded DNA in Endothelial Cells Is Mediated by NFκB and TNFα

**DOI:** 10.1371/journal.pone.0019910

**Published:** 2011-05-18

**Authors:** Suraj J. Patel, Rohit Jindal, Kevin R. King, Arno W. Tilles, Martin L. Yarmush

**Affiliations:** 1 Center for Engineering in Medicine and the Department of Surgery, Massachusetts General Hospital, and the Shriners Burns Hospital, Boston, Massachusetts, United States of America; 2 Harvard-MIT Division of Health Science and Technology, Harvard Medical School, Massachusetts Institute of Technology, Cambridge, Massachusetts, United States of America; 3 Department of Biomedical Engineering, Rutgers University, Piscataway, New Jersey, United States of America; University of Colorado Denver, United States of America

## Abstract

Endothelial cells represent an important barrier between the intravascular compartment and extravascular tissues, and therefore serve as key sensors, communicators, and amplifiers of danger signals in innate immunity and inflammation. Double stranded DNA (dsDNA) released from damaged host cells during injury or introduced by pathogens during infection, has emerged as a potent danger signal. While the dsDNA-mediated immune response has been extensively studied in immune cells, little is known about the direct and indirect effects of dsDNA on the vascular endothelium. In this study we show that direct dsDNA stimulation of endothelial cells induces a potent proinflammatory response as demonstrated by increased expression of ICAM1, E-selectin and VCAM1, and enhanced leukocyte adhesion. This response was dependent on the stress kinases JNK and p38 MAPK, required the activation of proinflammatory transcription factors NFκB and IRF3, and triggered the robust secretion of TNFα for sustained secondary activation of the endothelium. DNA-induced TNFα secretion proved to be essential *in vivo*, as mice deficient in the TNF receptor were unable to mount an acute inflammatory response to dsDNA. Our findings suggest that the endothelium plays an active role in mediating dsDNA-induced inflammatory responses, and implicate its importance in establishing an acute inflammatory response to sterile injury or systemic infection, where host or pathogen derived dsDNA may serve as a danger signal.

## Introduction

The endothelium serves as a key anatomic and functional barrier separating circulating cells and molecules of the vascular space, from the stroma and tissue-specific cells of solid organs. As such endothelial cells play a critical role in inflammation by sensing danger signals, and subsequently initiating the expression of cellular adhesion factors and the secretion of inflammatory cytokines [1], [2]. During an infection or sterile injury, pathogens and damaged host cells release danger signals such as lipopolysaccharides (LPS), peptidoglycans (PGN), high-mobility group protein-1 (HMGB1), and double stranded DNA (dsDNA) into circulation [3], [4], [5], [6], [7], [8]. Many of these danger signals have been shown to activate innate immune and inflammatory responses in endothelial cells [9], [10], [11], [12]. However, little is known about the direct effects of dsDNA on the vascular endothelium.

DNA has recently emerged as a potent activator of the innate immune system. Mammalian cells are exposed to dsDNA that is released from damaged host cells during tissue injury, or introduced by DNA viruses and intracellular bacteria during infection [13], [14], [15], [16], [17]. Many cytosolic receptors including Z-DNA binding protein 1 (ZBP1) and gamma-interferon-inducible protein 16 (IFI16) have been identified for sensing dsDNA [17], [18]. Signaling through these receptors triggers the activation of kinases such as TBK1 and Ikki, and the downstream phosphorylation of transcription factors IRF3 and NFκB [15]. Numerous investigations have revealed that dsDNA stimulation elicits a robust antiviral and inflammatory response in innate immune cells [15], [19], [20], [21]. Dendritic cells have been shown to secrete large amounts of interferon α/β, as well as chemokines IP10 and CCL5, in response to dsDNA [15]. In macrophages, dsDNA stimulation has been shown to trigger the robust production of proinflammatory cytokines IL1β and IL18 [20]. A recent study revealed that during sterile drug-induced liver injury, sinusoidal endothelial cells can also sense dsDNA from damaged hepatocytes and initiate an acute inflammatory response, however the mechanism remains unknown [14]. While endothelial cells are well known to respond to indirect stimulation by cytokines [22], their role as primary responders to direct stimulation by danger signals such as dsDNA is just recently emerging. The purpose of this study was to investigate the inflammatory response of endothelial cells to direct dsDNA treatment.

In this work we show for the first time that endothelial cells exposed to dsDNA trigger the activation of NFκB and MAPK pathways, including JNK and p38. This leads to increased expression of ICAM1, VCAM1 and E-selectin, and results in functional leukocyte adhesion. We demonstrate that endothelial sensing of dsDNA induces robust expression and secretion of TNFα, which mediates sustained secondary activation of the endothelium. Mice deficient in the TNF receptor are unable to mount an acute inflammatory response to DNA. Furthermore, both NFκB and IRF3 are required for the production of the TNFα in response to dsDNA. This study suggests a possible role for endothelial cells in mediating dsDNA-induced inflammatory responses, and implicates their importance in establishing an acute inflammatory response to systemic infection or sterile injury such as ischemia-reperfusion injury, where pathogen or host dsDNA may serve as a danger signal.

## Results

### Endothelial activation and leukocyte adhesion is triggered by dsDNA stimulation

**Figure 1:**
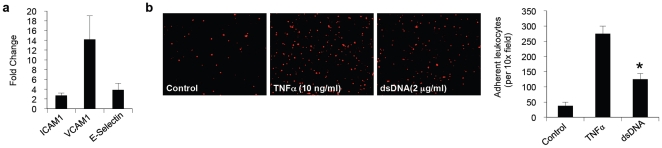
dsDNA induced activation of the endothelium. (a) Q-PCR for expression of ICAM-1, VCAM-1, and E-Selectin in endothelial cells after stimulation with dsDNA (2 µg/ml) for 4 hours. (b) Leukocytes adhesion to the endothelial cells stimulated with dsDNA (2 µg/ml), TNFα (10 ng/ml), or mock transfected with Lipofectamine alone (control) for 12 hours. (*P<0.05 compared to control).

Endothelial activation is an early event through which pathogen-associated molecular patterns induce inflammation during infection and tissue injury. Recent studies have established the role of dsDNA as a potent activator of the innate immune system [23]. For evaluating endothelial activation in response to DNA stimulation, we treated endothelial cells with synthetic B-form Poly(dA-dT)∶Poly(dA-dT) (hereafter referred to as dsDNA), a known intracellular DNA ligand [15]. dsDNA treatment resulted in increased expression of endothelial adhesion molecules ICAM1, VCAM1, and E-selectin (Figure 1a). The greatest increase in gene expression was observed for VCAM1 (Figure 1a). Adhesion molecules play a central role in leukocyte recruitment by regulating their attachment to the endothelium. To determine if dsDNA stimulation results in increased leukocyte adhesion, confluent monolayers of endothelial cells were treated with dsDNA, and then co-cultured with cell membrane dye-labeled peripheral blood leukocytes. TNFα stimulated endothelial cells were used as a positive control for measuring leukocyte adhesion under inflammatory conditions. dsDNA stimulation of RHMEC lead to significantly enhanced binding of leukocytes (3.5 fold), in comparison to Lipofectamine treated cells (Figure 1b). Our results indicate that dsDNA acts as a potent activator of the endothelium by increasing the expression of adhesion molecules and directly enhancing leukocyte adhesion.

### dsDNA-induced activation of NFκB and MAPK pathways

**Figure 2:**
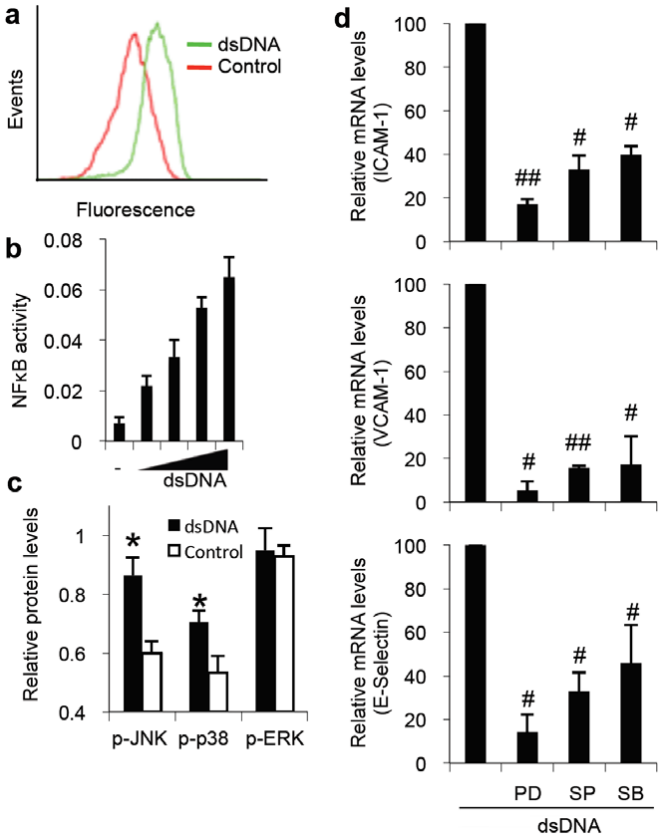
dsDNA activates NFκB and MAPK pathways, which modulate adhesion molecule expression in endothelium. (a) Fluorescence histogram of NFκB reporter clone of endothelial cells treated with Lipofectamine alone (control) or dsDNA (2 µg/ml) for 16 hours. (b) ELISA for NFκB activity in endothelial cells stimulated with a dose of dsDNA (0 to 4 µg/ml) for 6 hours. (c) Phosphorylated protein levels in endothelial cells stimulated with dsDNA (2 µg/ml) for 6 hours. (d) Q-PCR for expression of ICAM-1, VCAM-1, and E-Selectin in RHMECs after stimulation with dsDNA (2 µg/ml) for 4 hours in the presence or absence of PDTC (PD), SP600125 (SP), or SB202190 (SB), which are inhibitors of NFκB, JNK, and p38 MAPK pathways, respectively. (*P<0.05 compared to control, #P<0.05 and ##P<0.01 compared to dsDNA alone.)

NFκB and MAPK pathways are known to play an important role in regulating the expression of endothelial adhesion molecules [22]. Given that dsDNA stimulation leads to increased expression of adhesion molecules, we examined the activation of NFκB and MAPK pathways in response to dsDNA. For evaluating NFκB activation, a reporter clone of endothelial cells that synthesizes GFP in response to NFκB activation was utilized. dsDNA stimulation of NFκB endothelial cell reporters resulted in elevated levels of GFP in comparison to Lipofectamine stimulated cells, suggesting NFκB activation by dsDNA (Figure 2a). A nuclear ELISA for NFκB activation further confirmed that dsDNA induced dose-dependent activation of NFκB in endothelial cells after 6 hours of stimulation (Figure 2b). For evaluating MAPK activation, phosphorylated protein levels of JNK, p38, and ERK1/2 were measured in endothelial cells stimulated with dsDNA for 6 hours. Endothelial cells treated with dsDNA showed elevated levels of phosphorylated JNK and p38 in comparison to Lipofectamine treated cells (Figure 2c). However, levels of phosphorylated ERK1/2 were similar in both dsDNA stimulated and control cells (Figure 2c). These results suggest that dsDNA induces the activation of NFκB, JNK and p38 signaling pathways in endothelial cells, but not ERK1/2.

### Regulation of adhesion molecule expression by modulation of NFκB and MAPK pathways

We next sought to determine whether dsDNA induced activation of NFκB, JNK, and p38 pathways was required for increased expression of endothelial adhesion molecules. Endothelial cells were treated with dsDNA for 4 hours in the presence or absence of PDTC, SP600125, or SB202190, known inhibitors of NFκB [24], JNK [25], and p38 MAPK [26] pathways, respectively. Inhibition of NFκB, JNK, and p38 resulted in significantly reduced expression of ICAM1, VCAM1 and E-selectin, suggesting that all three signaling pathways are involved in dsDNA stimulated endothelial activation (Figure 2d). Inhibition of NFκB was most potent at reducing expression of these adhesion molecules (Figure 2d). Given that JNK and p38 MAPK are known activators of the AP1 family of transcription factors [27], our results suggest that transcription factors AP1 and NFκB may be required for dsDNA-induced endothelial activation.

### NFκB and IRF3 are required for dsDNA-induced TNFα production

**Figure 3:**
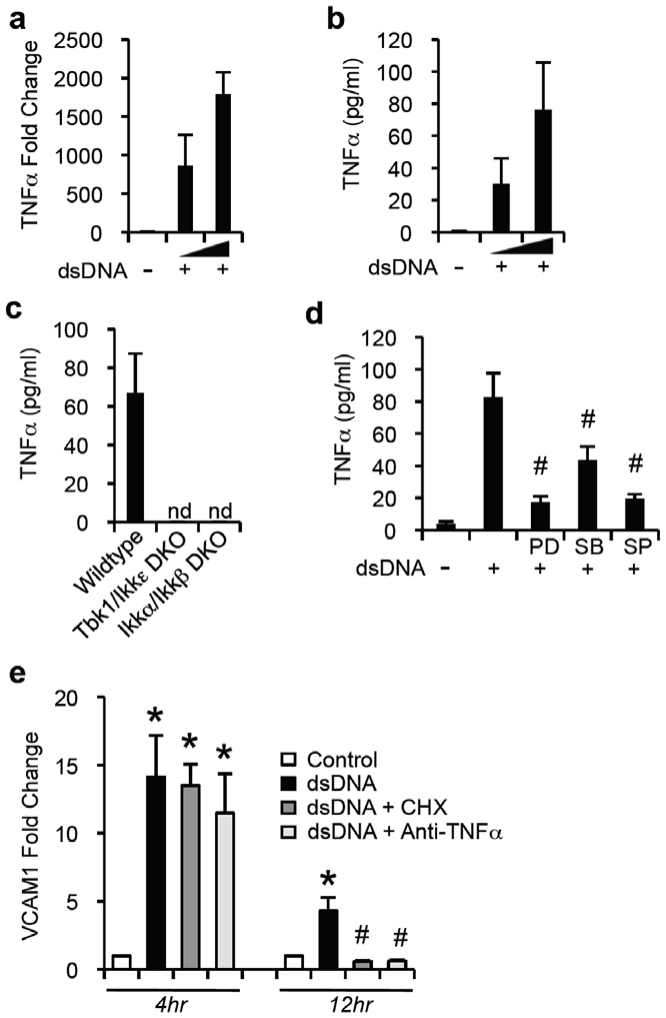
dsDNA mediated TNFα secretion for sustained secondary activation of the endothelium. (a) Q-PCR for expression of TNFα in endothelial cells after stimulation with dsDNA (.5 or 4 µg/ml) or mock transfected with Lipofectamine for 12 hours. (b) ELISA for TNFα in culture supernatant of endothelial cells stimulated with dsDNA (.5 or 4 µg/ml) or mock transfected with Lipofectamine for 24 hours. (c) ELISA for TNFα in supernatants of wildtype MEFs (WT), TBK1/IKKε DKO MEFs (TBK1/IKKε DKO), and IKKα/IKKβ DKO MEFs (IKKα/IKKβ DKO) stimulated with 4 µg/mL of dsDNA for 24 hours. (d) ELISA for TNFα in supernatants of endothelial cells after stimulation with dsDNA (4 µg/ml) for 24 hours in the presence or absence of PDTC (PD), SB202190 (SB) or SP600125 (SP) which are inhibitors of NFκB, p38 MAPK and JNK pathways, respectively. (e) Q-PCR for expression of VCAM1 in endothelial cells stimulated with dsDNA (1 µg/ml) for 4 or 12 hours, in the presence or absence of TNFα neutralizing antibody (+Anti-TNFa) or cycloheximide (+CHX). (*P<0.05 compared to control, #P<0.05 compared to dsDNA alone.)

**Figure 4:**
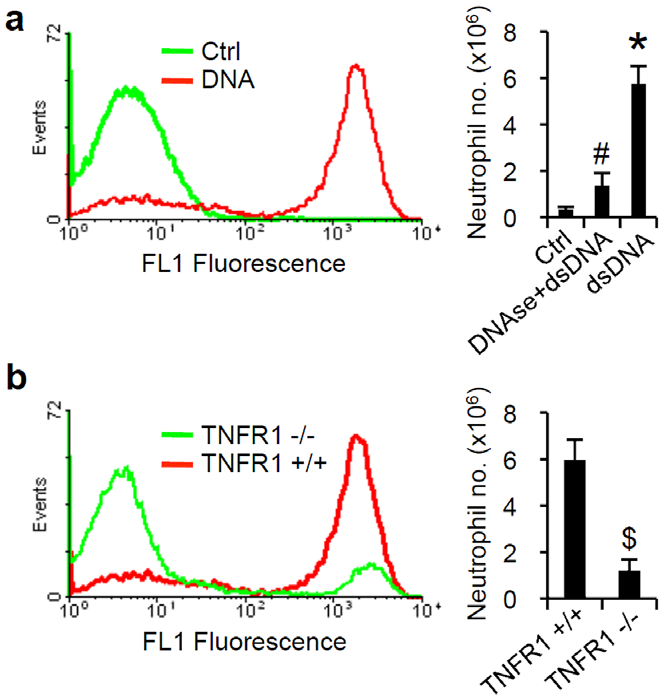
dsDNA induced acute inflammation depends on TNFα. (a,b) Representative fluorescence histograms of Ly-6G expression on peritoneal exudate cells (PEC) in mice injected 16 h earlier with saline (control), 2 µg/g of dsDNA, or 2 µg/g of DNAse digested DNA. The Ly-6G+ gate represents neutrophils. Neutrophil numbers in PEC of TNFR1+/+ and TNFR1−/− mice 16 h after i.p. challenge with 2 µg/g of dsDNA. Neutrophil numbers in PEC were determined by multiplying the total cell numbers by the percentage of Ly-6G+ cells. (*P<0.05 compared to control, #P<0.05 compared to dsDNA alone, α P<0.05 compared to TNFR1+/+ mice.)

TNFα is an important mediator of inflammation, as it acts on vascular endothelial cells to promote expression of adhesion molecules [28]. Having demonstrated mechanistically how direct stimulation of endothelial cells with dsDNA results in the expression of adhesion molecules, we next sought to investigate whether dsDNA induced TNFα production. dsDNA stimulation of endothelial cells resulted in upregulation of TNFα expression and robust secretion of TNFα into the culture supernatant, compared to Lipofectamine stimulation (Figure 3a,b). To determine the transcription factors necessary for TNFα production in response to dsDNA, we used transgenic knockout mouse embryonic fibroblasts (MEFs). Wildtype MEFs (WT) stimulated with dsDNA induced significant TNFα secretion after 24 hours (Figure 4c). Conversely, MEFs deficient in TBK1 and IKKε (TBK1/IKKε DKO), kinases necessary for IRF3 activation, failed to produce TNFα (Figure 3c). Similarly, dsDNA-stimulated MEFs deficient in IKKα and IKKβ (IKKα/IKKβ DKO), kinases essential for NFκB activation, also failed to produce TNFα (Figure 3c).To determine whether dsDNA-induced activation of NFκB, JNK, and p38 MAPK pathways was required for TNFα production, endothelial cells were treated with dsDNA for 24 hours in the presence or absence of known inhibitors of NFκB, JNK and p38 MAPK pathways. Inhibition of NFκB, JNK, and p38 MAPK significantly reduced TNFα production, although the reduction was more pronounced for NFκB and JNK inhibitors in comparison to p38 MAPK inhibitor. These results suggest that all three signaling pathways are involved in dsDNA stimulated TNFα production (Figure 3d). Together, these data suggest that dsDNA-induced TNFα secretion is dependent on both IRF3 and NFκB activation, and JNK signaling pathways.

We then sought to determine whether dsDNA-induced TNFα influences overall endothelial activation, since the expression of many adhesion molecules, such as VCAM1, is indirectly promoted by proinflammatory cytokines [22]. Using cycloheximide, a classic inhibitor of protein translation, we first clarified what portion of dsDNA-induced VCAM1 expression is from direct sensing of dsDNA and what part is from secondary indirect activation, requiring de novo protein synthesis. After 4 hours of dsDNA stimulation, VCAM1 expression, in the presence or absence of cycloheximide, was approximately equal (Figure 3e). However, after 12 hours of dsDNA stimulation, VCAM1 expression in the presence of cycloheximide was reduced to the level in unstimulated cells, whereas expression in the absence of cycloheximide remained high (Figure 3e). These data suggested that at an early time point, most of the measured VCAM1 expression was from direct sensing of dsDNA by endothelial cells, and that at later time points, VCAM1 expression was sustained by a secondary mechanism which required protein synthesis. Given the secretion of TNFα in response to dsDNA stimulation, we utilized a TNFα neutralizing antibody to investigate whether this secondary mechanism for sustained VCAM1 expression was due to the paracrine actions of TNFα. After 4 hours of dsDNA stimulation, VCAM1 expression, in the presence or absence of TNFα neutralizing antibody, was nearly similar (Figure 3e). In contrast, at 12 hours, VCAM1 expression in the presence of TNFα neutralizing antibody was curtailed to the level in unstimulated cells, suggesting that dsDNA-induced TNFα is required for sustained secondary activation of the vascular endothelium.

### dsDNA-mediated acute inflammation is dependent on TNFα

To evaluate the *in vivo* role of TNFα in acute inflammation triggered by dsDNA, we developed an *in vivo* model of dsDNA-induced inflammation. We injected complexed-DNA, as well as complexed-DNAse digested DNA, intraperitoneally (i.p.) into mice. After 16 hours, mice injected with dsDNA had abundant neutrophils in their abdominal cavities (Figure 4a), indicated by the staining of peritoneal lavage cells with the neutrophil marker Ly-6G. However, mice injected with DNAse digested DNA had minimal neutrophil infiltration (Figure 4a), in agreement with prior reports suggesting that DNAse digestion renders DNA inert to innate immune system. Mice deficient in the TNFα receptor (TNFR1−/−) also had significantly reduced neutrophil accumulation when injected with dsDNA, compared to abundant neutrophils in TNFR1+/+ mice (Figure 4b). Taken together, these results demonstrate that detection of dsDNA triggers acute inflammation dependent on TNFα signaling.

## Discussion

The endothelium exists at the interface between the vascular and tissue compartments, and is responsible for communicating danger signals during infection or sterile injury. Recently, dsDNA from pathogens and dying host cells was found to be a potent danger signal, capable of activating immune and inflammatory responses in many types of innate immune cells [13], [15], [16], [17]. In the current study we explore the consequences of directly stimulating endothelial cells with dsDNA. We show that dsDNA stimulation leads to expression and secretion of TNFα, as well as upregulation of surface adhesion molecules that facilitate leukocyte recruitment. These responses depend on activation of upstream MAP kinases JNK and p38, as well as activation of transcription factor NFκB. Furthermore, we provide evidence demonstrating that IRF3 and NFκB are both required for dsDNA-induced TNFα secretion, and show that TNFα is required for sustained secondary activation of the vascular endothelium.

The cellular response to dsDNA can be divided into two categories, a proinflammatory component mediated by the cytokines such as TNFα and IL1β, and an antiviral component mediated by type I interferons [20]. While the molecular pathways of the interferon response to dsDNA have been intensively investigated, the inflammatory arm has received comparatively less attention. TNFα and IL1β are arguably the most potent cytokines involved in inducing inflammation. They play a specific role in endothelial activation by inducing expression of cell surface adhesion molecules [22]. Investigations of dsDNA-induced inflammation have largely focused on the secretion of IL1β [20], [29]. Stimulation of cells with dsDNA triggers the robust secretion of IL1β by activating the inflammasome through the cytosolic DNA sensor absent in melanoma 2 (AIM2) and the endosomal DNA sensor TLR9, both of which are expressed in a cell type specific manner [29], [30]. These sensors bind dsDNA, and associate with inflammasome adaptor molecules for caspase-1 mediated secretion of IL1β, which can indirectly stimulate the endothelium [14], [29]. While the molecular details of dsDNA-induced IL1β secretion have been extensively investigated, the mechanism by which dsDNA triggers secretion of TNFα and its role in dsDNA-mediated inflammation remains relatively unexplored.

In this study, we focus on dsDNA-induced NFκB activation and TNFα secretion, and explore its ability to initiate and amplify a proinflammatory response in endothelial cells. We chose this cell type because of its unique location at the interface between tissue and vascular compartments, its likelihood of being exposed to danger signals, and because of its important functional role in recruiting immune cells to areas of injury. Endothelial cells are known to respond to indirect stimulation by proinflammatory cytokines commonly secreted in response to dsDNA [22], but their ability to directly sense dsDNA has not been examined. A recent study showed that free dsDNA released from apoptotic cells can directly stimulate endothelial cells to secrete IL1β and IL18 [14], however the mechanism remains unknown, and the subsequent activation of the endothelium unexplored.

In this report, we established that dsDNA sensing by endothelial cells leads to potent expression and secretion of TNFα. This is in agreement with prior studies using other cell types, which showed that undigested mammalian dsDNA induced TNFα secretion in macrophages [31], and enhanced expression of TNFα in dsDNA stimulated hepatocytes [32]. We also showed that dsDNA-mediated TNFα secretion was abrogated in MEFs deficient in kinases necessary for NFκB and IRF3 activation suggesting that both transcription factors are necessary for the response, which is analogous to LPS-induced TNFα secretion [33], [34]. Finally, we discovered TNFα as an essential mediator of dsDNA-induced acute inflammation, as mice lacking the TNF receptor exhibited a dramatic reduction in the inflammatory response to dsDNA stimulation *in vivo*. Although, it is important to note that eliminating the TNFα signaling did not completely abrogate the inflammatory response. Our studies did not resolve if the minor response observed in mice lacking the TNF receptor was due to direct induction of adhesion molecules by dsDNA or indirect contribution of some other cytokine(s). Dissecting the relative contributions of direct and indirect endothelial cell activation to this response, establishing the cellular source of TNFα, and determining possible involvement of other cytokine(s) will require further investigation.

Results from this study may have important implications in host defense and sterile injury. Direct sensing of dsDNA by the vascular endothelium suggests that endothelial cells may play an active role in mounting immune responses to systemic infection. In particular, DNA viruses such as cytomegalovirus (CMV), Epstein-Barr virus (EBV) and Herpes simplex virus-1 (HSV1) have been shown to infect the vascular endothelium resulting in dysfunction and injury that plays an important role in viral pathogenesis [10], [35], [36]. Endothelial cells may also be exposed to host dsDNA when cells die *in vivo* in the absence of infection due to sterile injury. During an ischemic injury, such as a myocardial infarction, damaged myocytes release genomic dsDNA locally into circulation [37] where it can stimulate a potent inflammatory response in endothelial cells, including a rapid influx of neutrophils to the site of injury. This sterile inflammation may contribute to the pathogenesis of acute ischemic injuries, as well as other forms of sterile injury including drug-induced toxicity. More studies are needed to better understand the precise role dsDNA plays in activating the endothelium during infection or sterile injury.

Another area of relevance for endothelial dsDNA-sensing is the response to gene therapy. The endothelium represents an attractive target for gene therapy because of its accessibility by intravenous infusion and its ability to communicate signals to the tissue compartment of solid organs. However, designing gene therapy vectors that minimize endothelial cell activation has proven to be challenging [38]. This could be related to the fact that gene therapy vectors are often derived from dsDNA-viruses, complexed plasmid dsDNA, or naked dsDNA, which can induce innate immune and inflammatory responses. Manipulating the immune and inflammatory responses of dsDNA-stimulated endothelial cells using knowledge of the underlying molecular machinery represents a unique opportunity to overcome these challenges.

## Materials and Methods

### Materials

Dulbecco's Modified Eagle Medium (DMEM), penicillin-streptomycin, and fetal bovine serum (FBS) were acquired from Invitrogen Life Technologies (Carlsbad, CA). Pyrrolidine dithiocarbamate (PDTC), SP600125, SB202190 were obtained from Tocris bioscience (Ellisville, MO). Synthetic polydeoxynucleotide, poly(dA-dT)∶poly(dA-dT) dsDNA, was purchased from Amersham Biosciences. MCDB-131-complete medium was obtained from VEC Technologies (Rensselaer, NY). Rat tumor necrosis factor-α (TNFα) was purchased from R&D Systems (Minneapolis, MN).

### Transfection of cells

Primary rat heart microvessel endothelial cells were acquired from VEC Technologies and maintained in MCDB-131 medium supplemented with 10% FBS, 10 ng/ml EGF, 1 µg/ml hydrocortisone, 200 µg/ml EndoGro, 90 µg/ml heparin, and 1% antimycotic solution. For transfections, endothelial cells were switched to DMEM media supplemented with 10% FBS, and 2% penicillin-streptomycin. dsDNA transfections were performed by using Lipofectamine LTX (Invitrogen) at a ratio of 1.5∶1 (volume/weight) with dsDNA as per manufacturer's protocol. For experiments involving the use of inhibitors, cells were pretreated with PDTC (5 µg/ml), SP600125 (20 µM), or SB202190 (10 µM) in DMEM for 1 h before dsDNA stimulation and during stimulation. For some experiments, endothelial cells were pretreated with a TNFα neutralizing antibody (2 µg/ml; R&D Systems), or cycloheximide (20 µg/ml; Sigma Alrich) for 1 hour before dsDNA stimulation and during stimulation.

Mouse embryonic fibroblasts (MEFs) were maintained in DMEM media supplemented with 10% FBS, and 2% penicillin-streptomycin. dsDNA transfections were performed as described above. Unless otherwise noted, all experiments were done three times.

### Real time polymerase chain reaction

RNA was extracted from cells using nucleospin RNA II kit (Macherey-Nagel Inc., Bethlehem, PA) according to the manufacturer's instructions. Quantitative Reverse Transcription PCR was performed using the Superscript III two-Step qRT-PCR kit purchased from Invitrogen (Carlsbad, CA). 500 ng of cellular RNA was reverse transcribed according to the manufacturer's directions. Real-time quantitative PCR was performed using the Stratagene (La Jolla, CA) MX5000P QPCR system. Each reaction was carried out with 10 ng cDNA and 0.6 µM primers. During amplification, the cycling temperatures were 95°C for 15 seconds, 57°C for 1 minute and 72°C for 30 seconds. The following primers were used for amplifying DNA: E-Selectin forward primer: CAACACATCCTGCAGTGGTC; E-Selectin reverse primer: AGCTGAAGGAGCAGGATGAA; ICAM-1 forward primer: CCTCTTGCGAAGACGA GAAC; ICAM-1 reverse primer: ACTCGCTCTGGGAACGAATA; VCAM-1 forward primer: TGAAGGGGCTACATCCACAC; VCAM-1 reverse primer: GACCGTGCAGT TGACAGTGA; TNFα forward primer: GTCTGTGCCTCAGCCTCTTC; TNFα reverse primer: GCTTGGTGGTTTGCTACGAC; β-actin forward primer: GTCGTACCACTGGCATTGTG; and β-actin reverse primer: CTCTCAGCTGTGGTGG TGAA. By using the comparative cycle threshold method, all data were normalized to endogenous reference gene β -actin and then compared with appropriate controls for calculation of fold change.

### TNFα ELISA

Supernatants from endothelial cells and MEFs were used to determine the amount of rat TNFα and mouse TNFα, respectively, that was secreted as measured by ELISA (R&D Systems) according to the manufacturer's protocol.

### Fluorescent Microscopy

Images were acquired using a Zeiss 200 M microscope (Carl Zeiss Inc., Thornwood, NY). The fluorescence images were captured using a CCD camera (Carl Zeiss Inc.) and Zeiss imaging software (Axiovision LE).

### Leukocyte adhesion experiments

Peripheral blood leukocytes were purified from heparinized peripheral blood of rat using Histopaque density gradient (Sigma) according to manufacturer's instructions. Leukocytes were labeled with CM-DiI (2.5 µg/ml) at 37°C for 10 minutes and then added to endothelial cells that were stimulated with dsDNA (2 µg/ml) or TNFα (10 ng/ml) for 12 hours. Leukocytes were allowed to adhere for 60 minutes at 37°C and then washed 3 times with PBS for removing non adhered cells. Fluorescence images were captured and analyzed using Image J software (National Institute of Health, Bethesda, MD) to estimate the degree of leukocyte adhesion to endothelial cells.

### Construction of NFκB reporter clone of endothelial cells

NFκB reporter plasmid consisted of multiple response elements upstream of destabilized green fluorescent protein gene that encodes for d2EGFP reporter protein [39]. The details describing the construction of NFκB reporter clone of endothelial cells are reported elsewhere [40]. Briefly, endothelial cells (2.5 million) were electroporated with the NFκB reporter plasmid (10 µg) using a BTX Electro Cell Manipulator 600 (Biotechnology and Experimental Research, San Diego, CA) at 280 V and 960 µF. Stably transfected clones were selected by addition of geneticin to a final concentration of 700 µg/ml. The clone that exhibited maximum shift in fluorescence upon stimulation with TNFα was used in experiments.

### Measuring NFκB and MAPK activity

Endothelial cells were stimulated with different dose of dsDNA ranging from 0 to 4 µg/ml for 6 hours. Nuclear extract was prepared from cells using Panomics Nuclear Extraction Kit (Affymetrix, Inc., Santa Clara, CA) as per manufacturer's protocol. Nuclear extracts were kept frozen in −80°C until further analysis. NFκB activity was determined by estimating the levels of NFκB p65 protein in the nuclear extract using ELISA kit (TransFactor NFkB p65 kit, Clontech, Mountain View, CA) according to manufacturer's instruction. Samples were normalized by total protein concentration of the nuclear extract, determined by Bradford reagent.

Endothelial cells were stimulated with dsDNA (2 µg/ml) for 6 hours in 96 well plate. Phosphorylated protein levels of JNK, p38 MAPK, and ERK1/2 were determined using cell based ELISA kit (RayBio Cell-Based ERK1/2 ELISA Sampler Kit, Ray Biotech Inc., Norcross, GA) as per manufacturer's instructions.

### Animals

C57BL/6 and TNFR1−/− mice were purchased from Jackson Laboratory. All animal protocols were approved by Massachusetts General Hospital Subcommittee on Research Animal Care.

### dsDNA-induced inflammation

dsDNA was complexed with LyoVec transfection reagent according to the manufacturer's protocol (Invivogen). DNAse I digested DNA was prepared by treating 100 µg of DNA with 5 U of DNAse I (Ambion) at 37 C for 30 minutes. DNAse was then heat inactivated, and the digested DNA was complexed with LyoVec. Mice were injected intraperitoneally with dsDNA (2 mg/kg) in .1 ml PBS, DNAse digested DNA (2 mg/kg) in .1 mL PBS, or PBS alone. At 16 hours after challenge, the numbers of neutrophils (Ly-6G+) in the peritoneum were evaluated as previously described [41]. For each condition, 5 animals were used.

### Statistical Analysis

Results are reported as mean ± standard deviation. Statistical analysis was performed using the Student's t-test, with P<0.05 considered significant.
